# Evaluating the impact of avian paramyxovirus type 1 infection in poultry at live bird markets in Nigeria: defining hurdles to sustainable agriculture

**DOI:** 10.1186/s12917-025-04508-2

**Published:** 2025-02-12

**Authors:** Abel B. Ekiri, Aliyu Wakawa, Scott M. Reid, Joe James, Craig Ross, Alexander M. P. Byrne, Thomas Lewis, Joshua Lynton-Jenkins, Kehinde Adebowale, Erik Mijten, Gabriel Varga, Ian H. Brown, Ashley C. Banyard, Alasdair J. C. Cook

**Affiliations:** 1 Department of Comparative Biomedical Sciences, School of Veterinary Medicine, University of Surrey, Guildford, GU2 7AL UK; 2 Department of Veterinary Medicine, Faculty of Veterinary Medicine, Ahmadu Bello University, Zaria, Nigeria; 3 Animal and Plant Health Agency (APHA-Weybridge), Department of Virology, Woodham Lane, Addlestone, Surrey KT15 3NB UK; 4 WOAH/FAO International Reference Laboratory for Avian Influenza, Animal and Plant Health Agency (APHA-Weybridge), Woodham Lane, Addlestone, Surrey KT15 3NB UK; 5 Zoetis-ALPHA Initiative, Zoetis, Zaventem, B-1930 Belgium

**Keywords:** Newcastle disease, Avian paramyxovirus, Virulent, Poultry, Nigeria, Live-bird markets, Africa

## Abstract

**Background:**

Infectious diseases including Newcastle disease (ND) impair poultry productivity and represent a significant burden to sustainable agriculture in Nigeria. This study aimed to investigate the active circulation and seroprevalence of Newcastle disease virus (NDV) caused by virulent forms of avian paramyxovirus type-1 (APMV-1) in poultry at live bird markets (LBMs) across Nigeria.

**Methods:**

A cross-sectional study of 18 LBMs was conducted within three states in Nigeria (Kano, Oyo, and Abuja). Paired swab and tissue samples (*n* = 413) were collected from birds on FTA cards and tested for APMV-1 using real-time reverse transcription polymerase chain reaction (rRT-PCR). A subset of rRT-PCR-positive samples were selected for whole genome sequencing based on the originating species (chicken, duck, geese), date, and market location to provide a broad range of isolates for characterisation. Blood samples (*n* = 405) were also collected from birds and the seroprevalence of APMV-1 antibodies was measured using an enzyme-linked immunosorbent assay (ELISA).

**Results:**

APMV-1 RNA was detected in 21.5% (89/413) of samples from LBMs by rRT-PCR. At least one APMV-1 positive sample was detected in 55.6% (10/18) of LBMs. The largest proportion of APMV-1-positive markets was in Kano (83.3%, 5/6), whereas the lowest was in Oyo (16.7%, 1/6). Assessment of genetic data demonstrated that genotype XIV.2 APMV-1 was circulating within Nigeria with the viruses detected clustering closely with other Nigerian isolates described previously. The seroprevalence of APMV-1 in birds was 45.9% (186/405) and 94.4% (17/18) of LBMs had at least one APMV-1 seropositive sample (i.e., with at least one APMV-1-antibody-positive bird). The LBMs in Kano had the lowest seroprevalence (88.3%, 5/6).

**Conclusions:**

This study demonstrated that APMV-1 continues to circulate in LBMs in Nigeria. LBM traders, poultry producers, and related industry and policy stakeholders should be aware of the occurrence of APMV-1 and how ND may negatively impact upon poultry production and the livelihoods of poultry farmers and LBM traders. Training initiatives aimed at improving the knowledge of APMV-1 infection and improvements in biosecurity practises and the role of disease mitigation through vaccination are required to reduce the impact of this threat to food security.

**Supplementary Information:**

The online version contains supplementary material available at 10.1186/s12917-025-04508-2.

## Background

In low-middle income countries (LMICs), poultry production is a key contributor to livelihoods and food security. In Nigeria, commercial and backyard poultry production provides a source of income across a wide transect of the population, where it serves as both an income supplement to salary-earners and as a main income source for the unemployed [1, 2]. Importantly, the poultry sector plays a gender specific role in affecting employment as women are often relied upon to undertake subsistence farming activities and maintain household-based livelihoods [1]. Based on data from 2017, the estimated poultry population in Nigeria was about 180 million birds, with the majority of birds being concentrated within the south-western part of the country [3]. The poultry industry continues to expand rapidly within Nigeria with chicken meat and egg production identified as critical food sources required to meet growing domestic demand [4]. However, critical to sector growth is the impact of avian viral diseases, which continue to significantly impact production [1, 5].

Avian paramyxoviruses are the causative agent of Newcastle disease (ND), with strains of avian paramyxovirus type-1 (APMV-1) (Subfamily Avulavirinae, Genus Orthoavulavirus) responsible for the virulent, velogenic form of the disease. In Nigeria, and other LMICs, ND is of high economic significance due to its impact on fowl production [6]; it is highly contagious [7] and often causes high morbidity and mortality rates in poultry [8]. The outcome of infection with a virulent APMV-1 depends upon both host and viral factors, although drivers for clinical disease outcomes remain poorly understood. The occurrence of mortality in birds depends on the virulence of the infecting APMV-1 strain. Mortality is strongly linked to the presence of multiple basic amino acids at a short motif within the fusion protein (F). Viruses with a cleavage site containing fewer basic amino acids and a leucine at position 117 are typically of low virulence [9]. In contrast, strains with a multi-basic amino acid cleavage site with a phenylalanine at position 117 can spread systemically within the bird, resulting in increased virulence [9, 10]. These ND strains may result in mortality of up to 100% in poultry [8]. Outbreaks can therefore have a devastating impact on poultry production, resulting in substantial financial losses for poultry producers, while also negatively impacting upon food security.

Several APMV-1 genotypes have been identified and linked to certain geographical areas and bird species. Since the first detection of ND in Nigeria in 1952 [11], numerous instances of ND have been detected in both domesticated and wild birds [12, 13]. These include vaccine spillover events (genotypes I and II) and isolates commonly associated with PPMV-1 (Pigeon paramyxovirus type 1, genotype VI, XX and XXI) which are common in wild and domesticated Columbiformes world-wide [14]. Some isolates appear to be geographically linked to West Africa, including genotypes XIV, XVII and XVIII, with the first isolates identified in West Africa in 2006/2007 (originally lineages 5 h, 5f and 5 g) [15, 16]. Since this initial identification, genotypes XIV and XVII appear to be the predominant genotypes detected in Nigeria, with these commonly found in both domestic poultry and in wild birds including pigeons, raptors, and a Malachite kingfisher (*Corythornis cristatus*) suggesting a dynamic interaction between viruses across the wild bird- poultry interface [12, 17]. Whilst directionality and evidence for viral exchange between different species is undefined, it is plausible that free range poultry come into contact with infected wild birds thus facilitating the maintenance and spread of AMPV-1 between domestic and wild avian populations [18].

Newcastle disease can present with or without clinical signs. Where NDV is present, clinical signs vary depending on multiple factors including virus strain, infected species, simultaneous presence of other infections and environmental stress [9, 19, 20]. However, the main clinical signs include respiratory signs (such as gasping, coughing, and sneezing), neurological signs (such as tremors, paralyzed wings and legs, twisted necks, circling, spasms, and paralysis), reduced egg production, and variable mortality [21]. APMV-1 infection may also occur asymptomatically; a study in apparently healthy commercial chickens in Nsukka (south-east Nigeria) showed that APMV-1 RNA was detected in 3.2% of apparently healthy chickens, highlighting that asymptomatic birds may act as a hidden source of the infection and spread the disease [22].

Several factors have been associated with the spread of APMV-1 in Nigerian poultry. A study of commercial and village flocks in Kaduna State reported that the distribution of APMV-1 was associated with age, breed, season, bird type and the management system of poultry [23], while in Kogi State, APMV-1 was observed more frequently in backyard and rural poultry farms than in those reared in an intensive system (62.9 *vs* 37.1%) [24]. Rainfall, humidity, altitude, and presence of live bird markets (LBMs) were also associated with AMPV-1 occurrence in a survey of poultry kept by farmers and traders in Gombe and Bauchi states [25].

Control strategies for ND include the prevention of APMV-1 introduction and spread by good biosecurity practices and vaccination [7]. In Nigeria, vaccination of fowl flocks against APMV-1 is routinely performed on commercial poultry farms [6]. However, as in many countries where the virus is endemic, vaccination alone is insufficient to control outbreaks and APMV-1 infection is detected with regularity across Nigeria in birds from backyard farms, rural free-range farms and live bird markets (LBMs). However, it is often unclear what proportion of sampled birds were previously vaccinated against APMV-1 in LBM, free-range, and backyard farms, and therefore, the seroprevalence data in such settings should be interpreted with caution [26]. For example, APMV-1 seroprevalence estimates ranged from 17% in local chickens in the Federal Capital Territory (FCT) Abuja [27], 35.8% in chickens from live bird markets (LBMs) in Zamfara State [28], 32.7% in free-range, domestic turkeys in Enugu State [29], 53.7% in village poultry species at poultry markets in Gombe State [30], and 25.6% in chickens from backyard farms, rural farms and LBMs in Kogi state [31]. Throughout Nigeria, LBMs are common and facilitate the trade and exchange of live fowl and as such provide an environment for disease spread as they congregate birds from many flocks, increasing risks of transmission between farms. As noted above, seroprevalence rates against APMV-1 in LBM fowl can be high and, in some cases, reportedly higher in chickens at LBMs than those in households (35.8 *vs* 26.8%, respectively) [28].

Despite the recognized risk of APMV-1 in poultry at LBMs, the evidence on circulating APMV-1 strains in poultry at LBMs and the role of LBMs in transmission is limited. This study aimed to characterise the circulation and impact of NDV in poultry from LBMs in and around three key metropolitan zones in Nigeria namely, Kano (Kano state, North-West), Abuja (FCT), North Central), and Ibadan (Oyo state, South-West). Alongside virus characterisation, a survey was conducted at the visited LBMs to characterise the biosecurity practices employed by market traders.

## Results

### Socio-demographic assessment of stall owners at selected LBMs

In total, 54 stall owners at LBMs completed the socio-demographic survey: 18 in Kano, 17 in Oyo, and 19 in the Federal Capital Territory (FCT) (Supplementary Table S1). Most stall owners were male (72.2%), had a secondary education or lower (81.5%) and were aged between 35–60 years (66.6%) (Supplementary Table S1). The gender balance of stall owners differed across the states (*P* < 0.001); all stall owners were male in Kano and the FCT (100%), whereas most stall owners in Oyo were female (88.2%). Nearly all owners were registered marketers with the state live bird market (96.3%) (Supplementary Table S1).

### Characteristics of the stalls and the practices of the stall owners at LBMs

Most of the market stalls (~ 80%) at LBMs were separated from other market sections (e.g., food, clothing) (Supplementary Table S2). Stall owners reported the presence of rodents or evidence of rodent activity at 59.3% of the market stalls at LBMs. In contrast observation or evidence of free-ranging wild birds was reported in less than a quarter (22.2%) of the market stalls at LBMs. In Oyo there was no evidence of rodent or wild bird activity reported at any of the stalls assessed in LBMs. In contrast, over 70% of the stalls in Kano and over 90% of the stalls in the FCT reported evidence of rodent activity in LBMs. Further, approximately a third of the stalls in Kano and the FCT were in markets where wild birds were present (Supplementary Table S2).

Across the three states, stall owners used both wooden (98.1%) and wire-mesh (88.9%) cages, with similar proportions between the states (Supplementary Table S2). Chickens were the most common poultry type sold (by over 90% of all surveyed stalls), followed by turkeys, guinea fowl and duck/geese. The stalls in the FCT were the most diverse in terms of the poultry types available for purchase. Moreover, stalls in the FCT were larger, with nearly 80% selling > 100 birds daily, compared to ~ 11% in Kano and ~ 6% in Oyo selling on a similar scale (Supplementary Table S2).

Stall owners sourced their birds mainly from village households (61.1%) and commercial farms (66.7%) but there were differences in sourcing of birds according to state. For example, all stalls in Kano (100%) and 63.2% of stalls in the FCT (63.2%) sourced birds from village households, compared to only 17.6% in Oyo (*P* < *0.001*). The main bird sources in Oyo were commercial farms but also other LBMs outside the state (Supplementary Table S2). Mobile sellers or middlemen were used to source birds by more than half of the stall owners in the FCT, while only 16.7% and 5.9% of owners did this in Kano and Oyo, respectively. Most stall owners at LBMs in the three states (74.1%) reported that they separated the newly bought and previously purchased birds; 100%, 72.2%, and 47.1% of stall owners in the FCT, Kano, and Oyo, respectively, always separated the birds.

Overall, a similar proportion of stall owners used commercial and locally compounded bird feed (around 67%); however, there were differences in commercial-feed use (*P* = *0.014*) and in locally compounded feed use (*P* < *0.001*) across the three states (Supplementary Table S2).

A substantial proportion of stall owners (48%) reported having 2–3 sick/dead birds per week; however, this mortality/sickness pattern was reported by over 60% of stall owners in Kano and the FCT but by only 18% of stall owners in Oyo (Supplementary Table S2). Over 70% of all stall owners reported that they isolated the sick birds at the stall. In Kano and the FCT this practice was reported by nearly all owners (94% and 100%, respectively) whilst the number of stall owners reporting isolation of sick birds was only 18% in Oyo (P < 0.001). Moreover, 84.2% of stall owners in the FCT reported the dead/sick birds to the veterinarian or veterinary clinic, while none and only 16.7% did this in Oyo and Kano, respectively (*P* < *0.001*). Most stall owners (70.4%) slaughtered sick birds for meat consumption. This was the principal means for disposal of sick birds in Kano (94.4%) and Oyo (76.5%), while in the FCT, slaughtering (42.1%) and burying/burning (36.8%) were practiced by a similar proportion of respondents.

Overall, a common practise to dispose of poultry waste (manure/faeces) included putting it in the waste dump site (39%) or selling it (43%). However, there were differences in the proportion of stall owners applying these practices across the three states; 82% of stall owners in Oyo reported putting the poultry waste in the waste dump site, while this was reported by only 22% and 16% of stall owners in Kano and the FCT, respectively (*P* < *0.001*). By contrast, 44% and 63% of stall owners in Kano and the FCT, respectively reported selling the poultry waste, while only 18% of owners in Oyo did so (*P* = *0.022*).

Less than a quarter of stall owners (22%) had their own transport and the vast majority (78%) used public vehicle (taxi, motorcycle) to transport poultry; these findings were similar across the three states (Supplementary Table S2). Most stall owners in Oyo (65%) reported transporting poultry from various sources together, while only 22% and 26% of owners reported doing so in Kano and the FCT, respectively (*P* = *0.016*). In the FCT, stall owners carried all the birds to be sold in baskets (95%) and/or in cages/crates (100%), while in Kano and Oyo, birds were also carried tied and piled loosely (by 44% and 29% of owners, respectively).

Most owners in Kano (78%) and all owners in the FCT reported that they always washed their hands after handling poultry, while less than half did so in Oyo (41%). All owners in the FCT and most owners in Oyo (65%) always cleaned the cages with soap and water after use but only 17% did so in Kano (Supplementary Table S2).

### Molecular assessment of APMV-1

**Fig. 1:**
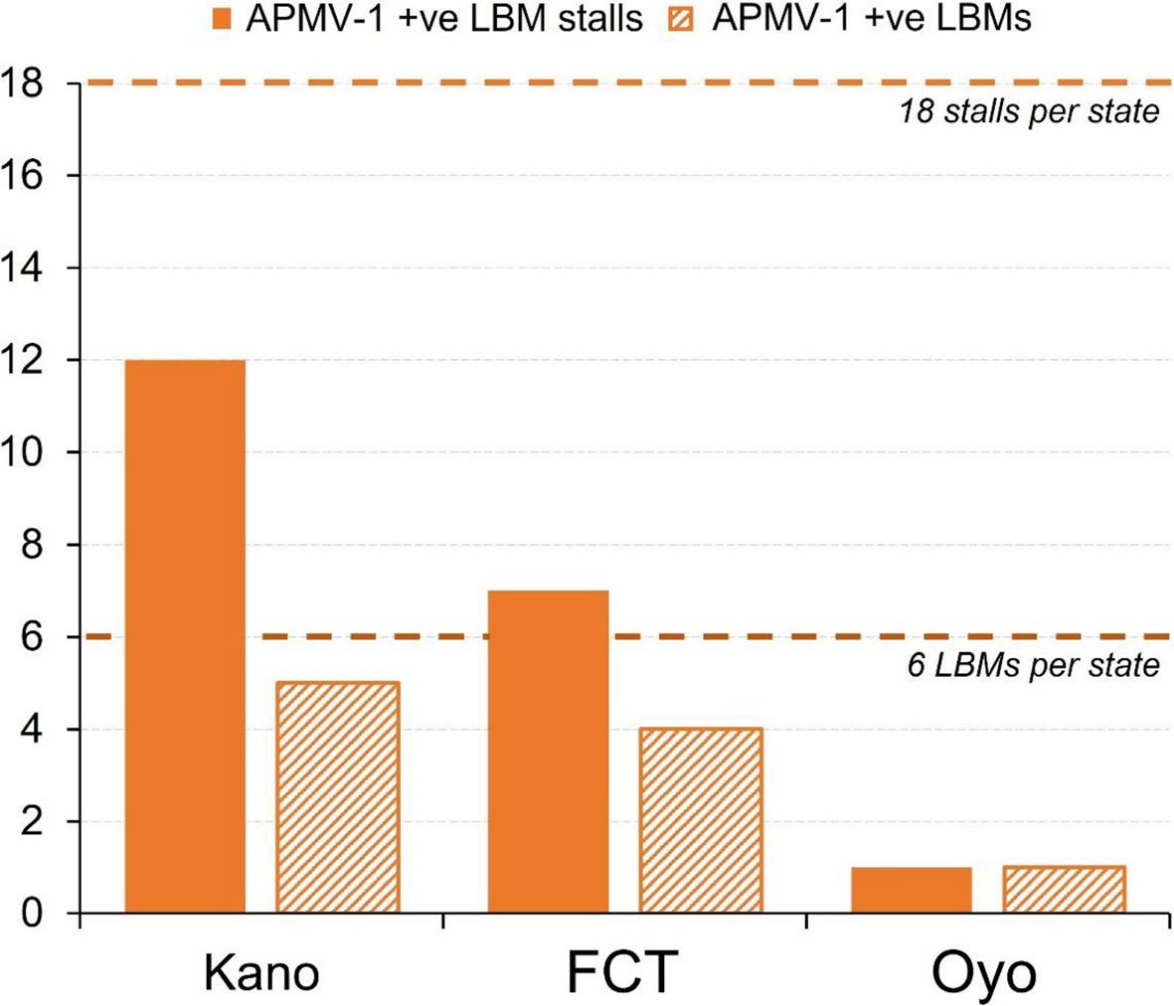
Number of APMV-1 + ve market stalls and markets for each metropolitan area (based on PCR data). Swabs and tissue samples were pooled by type for each stall. Stalls were recorded as positive where a positive result was obtained for at least one sample type and markets were recorded as positive if APMV-1 was detected in fowl from at least one stall

A total of 1086 swabs and tissue samples collected in the field across markets were subsequently processed in 352 sample pools (pooled by species and swab type, swab pools = 110 each, for cloacal and oropharyngeal; and tissue pools = 33 each, for liver, lung, proventricular, and spleen). All extracted RNA was assessed for the presence of APMV-1 nucleic acid. Of the 18 markets across the three states, 10 (56%) had at least one market stall positive for APMV-1 (i.e., defined as APMV-1 RNA positive markets for the current study). The largest proportion of APMV-1-positive markets was in Kano (83%), whereas the lowest was in Oyo (17%) (Fig. 1).

Collating the positive results obtained at each market stall revealed significant differences in APMV-1 positivity detected between the three metropolitan areas (*P* < 0.001); the highest proportion of positive stalls was in Kano, whereas the lowest was in Oyo (67% *vs* 6%, respectively; Fig. 1). Overall, a higher proportion of the pooled oropharyngeal swabs tested positive for APMV-1 RNA (18%) compared to the pooled cloacal swabs (9%, *P* = *0.05*; Table 1). Across the three metropolitan areas, a higher proportion of the pooled oropharyngeal swabs tested positive for APMV-1 RNA in Kano (36%) compared to FCT (16.7%) and Oyo (2.6%) (*P* = < 0.01; Table 1). No significant difference was detected in positivity between swabs obtained from chickens versus waterfowl (Table 1). In the pooled tissue samples, the proportion of APMV-1 positive pools detected across tissue types was liver = 27%, lung = 30%, proventricular = 27%, and spleen = 27%.

**Table 1:** APMV-1 rRT-PCR results from cloaca and oropharyngeal swabs separated by sampling location (metropolitan area) and bird type

APMV-1 rRT-PCR swab screening				
	**Total**^a^	**Positives**	**Negatives**	P^*^
	N	n (%)	n (%)	
**Location (State)**^b^				
**Cloacal swabs**				NA
Kano	36	9 (25%)	27 (75.0%)	
FCT	36	1 (2.8%)	35 (97.2%)	
Oyo	38	0 (0%)	38 (100.0%)	
**Oropharyngeal swabs**				< **0.01**
Kano	36	13 (36.1%)	23 (63.9%)	
FCT	36	6 (16.7%)	30 (83.3%)	
Oyo	38	1 (2.6%)	37 (97.4%)	
**Bird type**^c^				
**Cloacal swabs**				0.75
Chickens	72	7 (9.7%)	65 (90.3%)	
Waterfowl	38	3 (7.9%)	35 (92.1%)	
**Oropharyngeal swabs**				0.63
Chickens	72	14 (19.4%)	58 (80.6%)	
Waterfowl	38	6 (15.8%)	32 (84.2%)	
**Totals**				< **0.05**
**Cloacal swabs**	110	10 (9.1%)	100 (90.9%)	
**Oropharyngeal swabs**	110	20 (18.2%)	90 (81.8%)	

*APMV-1* Avian paramyxovirus; *rRT-PCR* real-time reverse transcription polymerase chain reaction; *N/A* not applicable

*P-values are from Chi-square tests

^a^Totals pertain to the number of sample pools tested, details on sample pooling are provided in the methods

^b^Difference in positivity between locations was tested using all sampling results (Fig. 1)

^c^Bird type definitions: chickens (i.e., local, broiler, layers & cockerels) and waterfowl (i.e., ducks and geese)

#### APMV-1/Avian paramyxovirus serotype-1 pathotyping and genetic sequencing

**Fig. 2:**
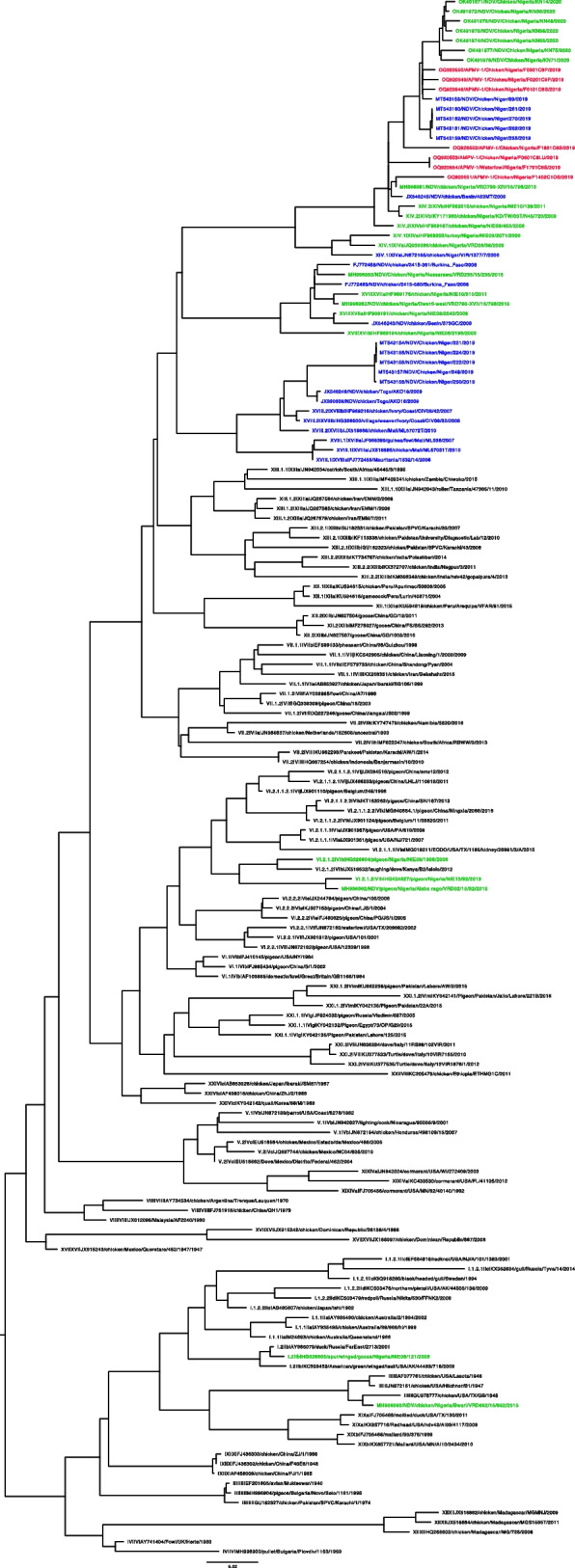
Maximum-likelihood phylogenetic tree of the APMV-1 F-gene segment. Sequences detected in Nigeria during the current study are highlighted in red, historic samples from Nigeria are in orange, and samples collected from the wider West Africa region are in blue. Sequences were aligned using MAFFT v7.48790 and the phylogenetic tree inferred using GTR + I + G model with ultrafast bootstrap node support and the sequences were mid-point rooted

A selection of samples (*n* = 10, Supplementary Table S3), based on location of detection and initial Ct values, were whole genome sequenced. Phylogenetic analysis demonstrated that all the viruses clustered with other samples previously detected in Nigeria within genotype XIV.2 (Fig. 2). All sequences had an avirulent cleavage site (GRRRKR/F) confirming that APMV-1 was circulating in birds in the region.

### Serological assessment

Blood samples were collected from 421 birds across 18 markets. Of these, the serum samples of 405 birds were tested for APMV-1 antibodies using an ELISA (ProFLOK® AIV ELISA kit, Zoetis). In total, 46% (n = 186) of birds were seropositive for APMV-1 specific antibodies (Table 2).

**Table 2:** Summary of APMV-1 ELISA results by state, bird type, number of birds at stall, signs of illness, and mortality pattern at stall

APMV-1 ELISA test				
	**Total**	**Positive**	**Negative**	P^*^
	N	n (%)	n (%)	
**All birds**	405	186 (45.9%)	219 (54.1%)	N/A
**Location (State)**				** < 0.001**
Kano	136	43 (31.6%)	93 (68.4%)	
Oyo	134	79 (59.0%)	55 (41.0%)	
FCT	135	64 (47.4%)	71 (52.6%)	
**Bird type**^a, b^				0.804 ^1^
Chickens (e.g., local, broilers, layers, cockerels)	313	147 (47.0%)	166 (53.0%)	
Waterfowl (e.g., ducks, geese)	85	38 (44.7%)	47 (55.3%)	
**Average number of birds on the stall**^a^				0.265
< 50 birds	73	37 (50.7%)	36 (49.3%)	
50–100 birds	183	77 (42.1%)	106 (57.9%)	
> 100 birds	142	71 (50.0%)	71 (50.0%)	
**Apparent signs of illness observed by interviewer**^c^				** < 0.001** ^1^
No	259	140 (54.1%)	119 (45.9%)	
Yes	132	45 (34.1%)	87 (65.9%)	
**Apparent signs of illness observed by stall owner**^a^				0.443 ^1^
No	63	26 (41.3%)	37 (58.7%)	
Yes	335	159 (47.5%)	176 (52.5%)	
**Mortality pattern on stall (average dead birds/week)**^a^				** < 0.001**
No deaths	15	12 (80.0%)	3 (20.0%)	
Rarely (up to 1/month)	13	8 (61.5%)	5 (38.5%)	
1–3/week	132	49 (37.1%)	83 (62.9%)	
2–4/week	119	54 (45.4%)	65 (54.6%)	
5–7/week	44	31 (70.5%)	13 (29.5%)	
10/week or more	75	31 (41.3%)	44 (58.7%)	
**Market APMV-1 status (RT-PCR)**				
Positive n (%)	10	9 (52.9%)	1 (100%)	N/A
Negative n (%)	8	8 (47.1%)	0 (0%)	

*ELISA* enzyme-linked immunosorbent assay; *FCT* Federal Capital Territory; *N/A* not applicable; *APMV-1* avian paramyxovirus, *RT-PCR* reverse transcription polymerase chain reaction

^*^P-values are from Chi-square tests

^1^*P*-value is from a Chi-square test after Continuity Correction for comparisons in 2 × 2 tables

^a^Survey data were available for n = 398 birds that were tested for APMV-1

^b^The results for the bird sub-types are shown separately in Additional file 3

^c^Data were available for n = 391 birds that were tested for APMV-1

**Fig. 3:**
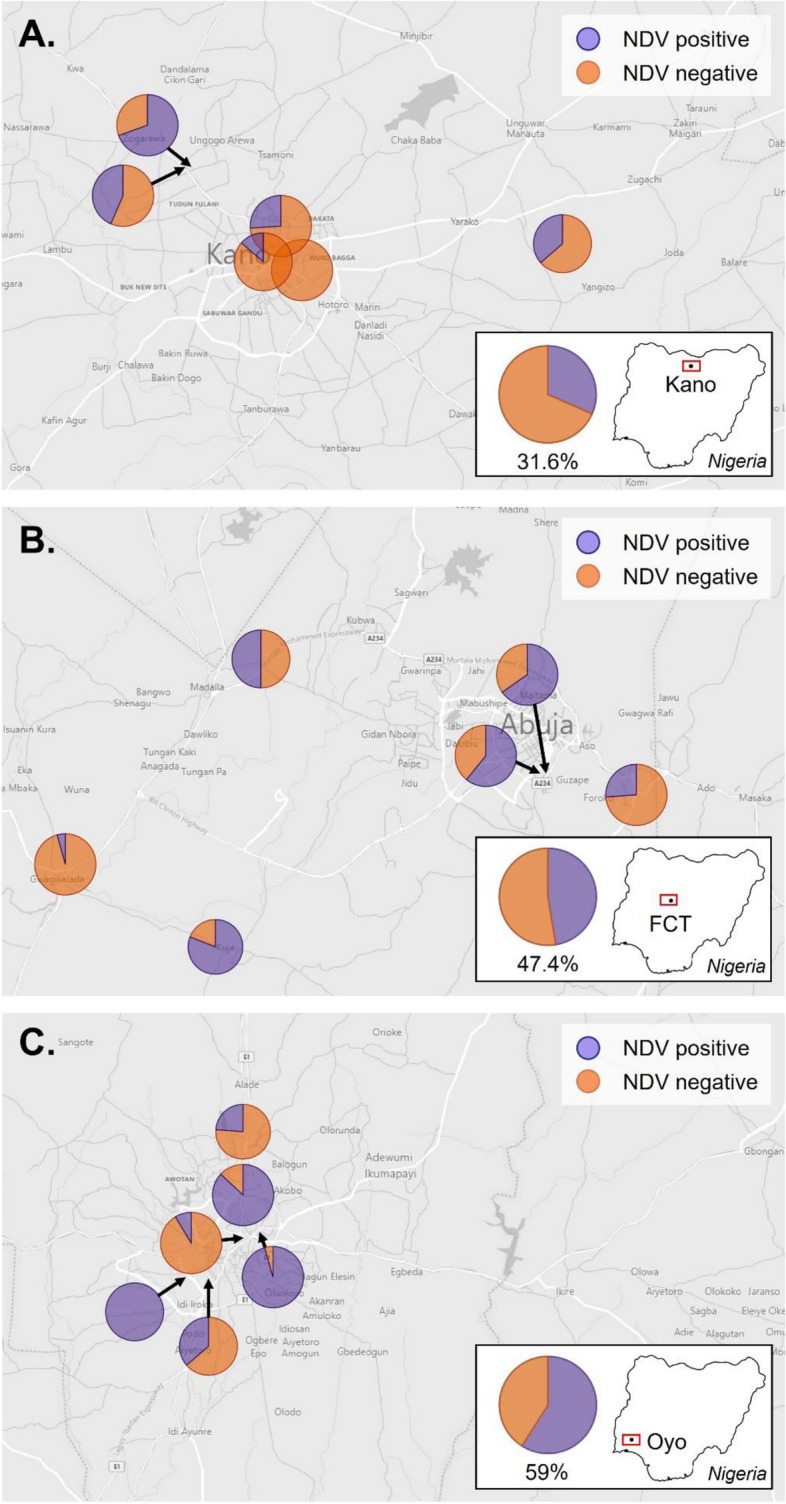
Map detailing the locations of live bird markets (LBMs) across the three survey regions within Nigeria and the seroprevalence for NDV detected at each market. The map was created by authors. Also included inset is the average seroprevalence for the given region: **A** LBMs in Kano, **B** LBMs in the FCT, and **C** LBMs in Oyo

There was a significant difference in the proportion of APMV-1-seropositive birds across the three states (*P* < 0.001), with higher proportions of seropositive birds in Oyo (59%) and the FCT (47%) than in Kano (32%) (Table 2, Fig. 3). There was no difference in the proportion of APMV-1-positive birds by bird type (chicken *vs* waterfowl: 47% *vs* 45%; *P* = 0.804) (Table 2). The average number of birds present at individual stalls was not significantly different between APMV-1 seropositive and negative birds (*P* = 0.265) (Table 2). There was a significant difference in the proportion of APMV-1 seropositive birds between birds with and without apparent signs of clinical disease (54% *vs* 34%; *P* < 0.001). There was a higher percentage of APMV-1-positive birds at the stalls with low reported mortality (no deaths and up to one death per month; between 62–80%) but also at the stalls with high mortality (5–7 deaths/week; ~ 71%) (Table 2).

#### Market APMV-1 status based on serology antibody data

Of the 18 markets, 17 markets (94%) were seropositive for APMV-1 (i.e., the markets had at least one APMV-1-antibody-positive bird) (Fig. 3). All markets in Oyo and the FCT were APMV-1-antibody-positive (100%) and the lowest proportion of APMV-1-antibody-positive markets was in Kano (88%). When market APMV-1 positivity was based on both serology antibody data (sero-prevalence of pathogen exposure) and rRT-PCR data (current pathogen circulation); 53% of the markets were APMV-1 positive by both methods and no markets were negative (Table 2).

## Discussion

This cross-sectional study of 18 LBMs in three states in Nigeria (Kano, Oyo, and the FCT) has demonstrated that strains of APMV-1 were circulating at the study sites at the time of sampling. These strains, detected in traded poultry and waterfowl, were phylogenetically similar to strains previously reported from Nigeria. Prevalence of APMV-1 at LBMs detected by RT-PCR varied between states, with prevalence highest in Kano and lowest in Oyo. This contrasted with the serological results, where Oyo state had the highest rate of positivity. Identifying LBMs and related areas of high transmission risk for APMV-1 will further our understanding of the spread of ND within Nigeria and help inform the targeting of biosecurity efforts.

The LBM survey captured disparate levels of awareness of NDV among LBM stall owners with considerable between-state variation in both hygiene practices (e.g., cleanliness and disinfectant use) and risk reduction (e.g., separation of stock and isolation of sick birds) recorded. Stall owners in the FCT, the federal capital, described adhering to the best hygiene practices and the highest rate of disease reporting to veterinarians, while stall owners in Kano and Oyo performed poorly in this regard. The reasons for the differences between LBMs across states are not clear. It is important to note the LBM survey findings were self-reported, as such are prone to potential reporting bias, and therefore should be interpreted with caution. Overall, most stall owners at LBMs (74.1%) in the three states reported that they separated the newly bought and previously purchased birds, with Oyo state performing slightly poorly compared to Kano and FCT. The reasons for the differences between LBMs across states are also not clear. However, this finding suggests there is a level of awareness of the risk of introduction of disease in birds at LBMs and provides a foundation on which to build risk mitigation strategies targeting LBM marketers. In Nigeria, LBMs are important hubs for the exchange and congregation of poultry stock, providing key opportunities for viral transmission [32]. Previous outbreaks of high pathogenicity avian influenza (HPAI) encouraged the development of interventions to improve biosecurity at LBMs. At the national level, legislative constraints on poultry production now relate to both commercial farms and LBMs. For example, the revised Animal Diseases Control Act (2022) requires state registration of poultry farms, hatcheries, and LBMs. It also requires farm owners to maintain the vaccination of stock and good hygiene practices [33]. Educational programmes have also been introduced to improve LBM vendor awareness of viral disease [34]. The study findings suggest that educational programmes targeting LBM marketers should consider the between-state variation in awareness of hygiene and risk reduction practices as well as gender differences among LBM marketers, while leveraging the benefit of easy access to LBM marketers as most are registered at the state level.

Overall, APMV-1 was circulating in more than half (56%) of the LBMs investigated (i.e., these markets had at least one APMV-1-positive sample from an rRT-PCR test). Detected strains clustered within genotype XIV.2, most closely related to previously detected Nigerian isolates, indicating that endemic strains specific to domestic fowl were transmitted nationwide with no evidence of recent introductions from other wild avian species. Within Nigeria, avirulent Genotype I.2 and genotypes VI.2 and XXI, commonly associated with PPMV-1 have also been identified in wild birds (spur-winged geese and pigeons respectively) [12, 14, 16], however, these were not found to be circulating within the markets sampled here. Genotypes detected herein contrast with Eastern and Southern Africa where virulent Genotype VII APMV-1 viruses have been isolated in multiple locations throughout this region of the continent [35–38].

Among the three metropolitan areas, Kano had the highest overall proportion of APMV-1-positive samples (40%) and LBMs (83%). This result is concordant with a previous survey of LBMs in the state of Kano, where it was found that 27% of 48 tested swabs were positive for APMV-1 (38% of 24 oropharyngeal swabs and 17% of 24 cloaca swabs) [39]. In contrast, in the present study, prevalence of APMV-1 was significantly lower in both the FCT (14%) and Oyo (< 1%). Sampling in the present study was conducted in November, at the onset of the dry season. Previous studies have highlighted associations between APMV-1 prevalence and precipitation, suggesting outbreaks of disease are more common during drier climatic conditions [40–43]. In the present study, sampling spanned the latitudinal extent of Nigeria, from the state of Oyo in the wetter south (Guinea climatic zone) to Kano in the drier north (Sahel climatic zone) [44]. The pattern of prevalence therefore supports a potential association between climate and APMV-1 prevalence, however, whether this association results from viral (e.g., environmental persistence) or host factors (e.g., seasonal stress on immune response) requires further study. Additional to this association with climate, the LBM marketer survey revealed state specific patterns in the sourcing of market stock. In Kano and FCT, 100% and 63.2%, respectively, of responding stall owners sourced stock from village households. In contrast, stall owners in Oyo sourced from village households at the lowest rate (17.6%) and most stall owners sourced from commercial farms. In Nigeria, vaccination against NDV is required by law on commercial poultry farms and therefore stock originating from this source may be less prone to infection. Nationwide data on ND vaccination are lacking but general vaccine use against endemic diseases is reportedly > 80% on farms across Nigeria [45].

Differences in APMV-1 detection were not observed between the tissue types screened, however, from the swabs a higher rate of detection was observed from oropharyngeal swabs. Of 110 swab pools, only 1 of the cloacal swab sample pools failed to be detected from the paired oropharyngeal swab pool, while an additional 9 swab pools tested positive using oropharyngeal swabs versus cloacal. Although this would suggest improved detection in oropharyngeal swabs, several studies have reported contrasting results on detection accuracy dependent on swab type [46–48]. For example, in contrast to the present study, a study of poultry from LBMs and backyard farms in Indonesia found that a higher proportion of APMV-1-positive samples (by rRT-PCR) were obtained from cloacal swabs than oropharynx swabs (29% vs. 7% respectively) [48]. Swab-target dependent detection in epidemiological studies likely results in variation due to the progression of infections resulting from both the timing of sampling and the virus strain involved, both factors have been shown to alter viral shedding profiles from the oral and faecal routes [49]. Additionally, the presence of PCR inhibitors may vary with target site and influence downstream detection in the absence of additional purification steps [50]. Finally, the use of FTA cards for detecting viral RNA versus directly collecting swabs or tissues in transport media may affect downstream detection. Evaluation of the effectiveness of FTA cards for detection of APMV-1 was beyond the scope of this study but has previously been demonstrated [51]. FTA cards are widely used for sample storage for detection of viral pathogens and can provide good sample preservation, including at high ambient temperatures [52, 53]. To minimise variation in use of FTA cards in the present study, the field study teams were trained in their application prior to sample collection. Within each state, the same study team collected samples at each site. As such, the variation seen here more likely relates to sample type, and these results emphasise the value in obtaining swabs from both orifices to improve the likelihood of virus detection and correct diagnosis.

Serology was utilised to detect previous host exposure to APMV-1 antigens either through vaccination or prior exposure and survival following avirulent APMV-1 infection. APMV-1 antibodies were detected in 45.9% of blood samples and in 94.4% of the LBMs. The seroprevalence of APMV-1 (measured using ELISA and haemagglutination inhibition test) has been reported to vary between 17 and 73% in Nigerian fowl, with samples measured in different years and localities [26, 28, 29, 54]. Vaccination against NDV is performed routinely on commercial poultry farms with layer and broiler flocks in Oyo and Kano states [6]. Different NDV vaccines are used, and the strains covered by the vaccines include Komarov (mesogenic), Unspecified mesogenic, R2B (mesogenic), LaSota (lentogenic), B1 (lentogenic), and VH (lentogenic) [6]. This confounding factor could explain both the variation in seroprevalence between studies in similar localities, and the discrepancies between prevalence detected by PCR and serology [26]. In the FCT, for example, we report a seroprevalence of 47% and previous studies have reported APMV-1 seroprevalence in the FCT at 17% (~ 2014 [27]), 57% (~ 2014 [55]), and 63.5% (~ 2016 [56]), with only one of these studies accounting for host vaccination status [55]. In addition, in the present study, Oyo had the highest seroprevalence of APMV-1 antibodies among the three states (59%) but less than 1% prevalence by rRT-PCR. Low viral prevalence and high seroprevalence could indicate that birds in Oyo were previously exposed to a significant outbreak of APMV-1 or alternatively that vaccination is more effectively applied in this region. Oyo state has the highest density of commercial poultry production in Nigeria [57]. As noted above, stall owners from Oyo reported lower rates of stock sourcing from village sellers compared to owners from the other two states, therefore a higher proportion of stock was sourced from commercial farms, increasing the likelihood of prior vaccination. Additionally, stall owners' perceptions of mortalities in their stock supported the epidemiological results, as a lower proportion of stall owners in Oyo reported sick or dead birds compared to either Kano or the FCT.

The circulation of APMV-1 will impact upon productivity even in the absence of clinical disease or mortalities. Profiling shedding characteristics across varied species at different clinical stages is important but is often not possible within field settings. In the current study, there was a significant difference in the proportion of APMV-1 seropositive birds between birds with and without apparent signs of clinical disease. Further investigation is required to determine if birds with or without observed clinical signs were more likely to test seropositive for APMV-1 antibodies. Previously, APMV-1 has been isolated in 3.2% of clinically healthy chickens in south-east Nigeria [22]. This is an important consideration as the infected poultry can act as a hidden source of infection. Biosecurity at different sites, and bio-secure practises undertaken by different bird producers will also impact upon the spread of infection. To truly make an impact upon the incursion of these viruses within different settings, a full assessment of the network within which live bird trade occurs and weaknesses within those networks where virus may get into systems is required. The present study illustrated that these practices vary between LBMs across Nigeria. Further research of vaccination practises within the country would elucidate the epidemiological picture of APMV-1 and provide evidence to develop a framework for preventing incursions of avian viral diseases. The present study confirms that LBMs continue to offer opportunities for the transmission of avian viruses and highlights a need for further investigation to determine if birds arrive at market infected or acquire the infection at the market.

## Conclusion

The current study found that APMV-1 was circulating in LBMs in the study sites in the three states of Nigeria at the time of sampling, with more than half of LBMs classed as APMV-1-positive. APMV-1 is highly contagious and can result in outbreaks in poultry. LBM traders should therefore be aware of APMV-1, understand how APMV-1 is transmitted, and the biosecurity measures required to prevent and minimise its spread. The survey revealed considerable disparity in this understanding between the three states surveyed. The virulent form of APMV-1, ND can have a devastating economic impact on poultry production and the poultry industry, and it poses risks for food security. LBM traders should be made aware of these risks to their income and food security; this could serve as a motivation to adopt and adhere to biosecurity measures. Training and education of LBM traders about adequate biosecurity practices can help lower the risk of transmission of APMV-1 in these settings. Ensuring access to vaccination of village poultry could reduce ND prevalence in areas which predominately rely on this source of stock e.g., in Kano state.

## Methods

### Study approach

**Fig. 4:**
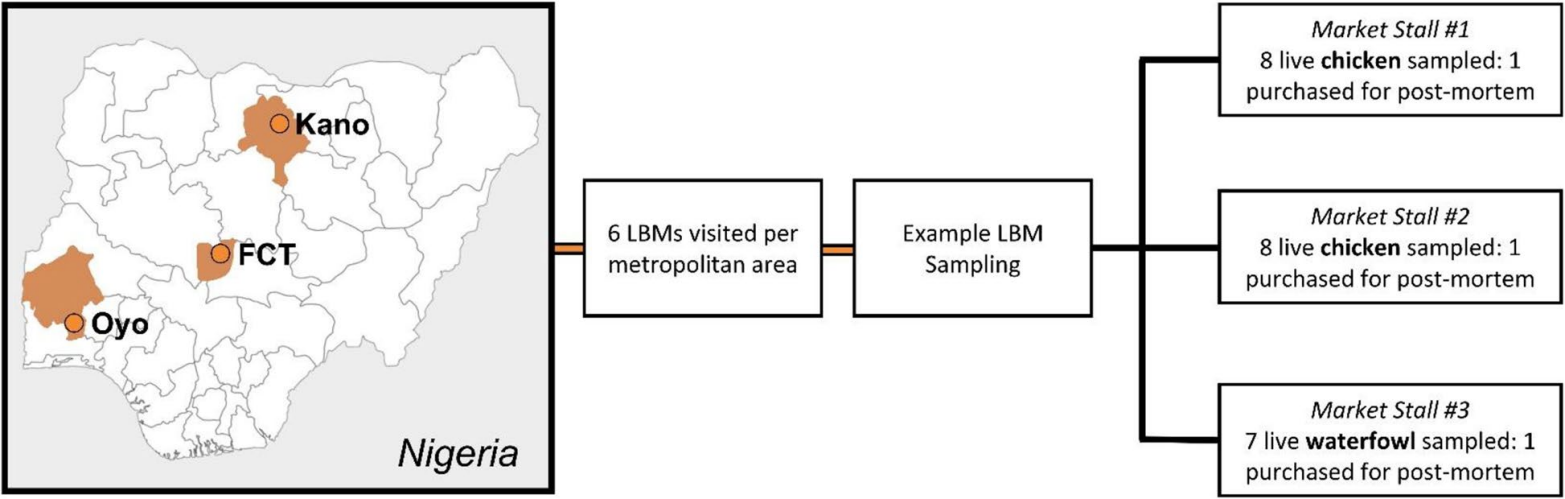
Sampling approach for LBMs in the three states which was centred around the following metropolitan areas: Kano, Kano; Abuja, FCT; and Ibadan, Oyo. Abbreviations: LBM, live bird market. The map was created by authors

This study was designed to both investigate APMV-1 circulation and seroprevalence in birds sold at live bird markets in Nigeria and to characterise socio-demographics of marketers/stall owners and biosecurity measures implemented at LBMs. Three states located in three geopolitical zones of Nigeria namely, Kano (North-West), the FCT (North Central), and Oyo (South-West) were selected for inclusion in the study. Within each selected state, a list of available LBMs was obtained and a representative sample of 6 LBMs was randomly selected (Fig. 4). Each state is divided into administrative units called Local Government Areas (LGAs). In LGAs with more than one LBM, a single LBM was selected at random.

The following inclusion criteria were also considered: LBMs registered with state LBM associations, LBMs that operate daily, live-bird marketers that have birds in stalls/cages at the time of sampling and have registered with LBMs association, and LBMs selling chicken, ducks, waterfowl and geese were eligible. The exclusion criteria included: areas with highly unstable security at the time of the study and marketers who were unwilling to allow the study team to sample birds were excluded.

### Sample size determination of birds

The sample size of birds for sampling was estimated based on an expected sero-prevalence of 9.5% [58] for poultry in LBMs, a desired absolute precision of 5%, and a confidence interval of 95%, as described previously [59]. A minimum sample size of 133 birds from LBMs was estimated and 5 birds were added to account for attrition, resulting in a minimum of 138 birds sampled from LBMs per state [total for study: 414 birds (138 × 3 states)]. At each LBM, 23 birds were selected, giving a total of 138 birds (23 × 6 LBMs) sampled in each state. At each selected LBM, 3 poultry stalls/marketers were purposively selected based on the type of bird species sold (as specified in the inclusion criteria above) and willingness to participate in the study.

At each LBM, two stalls with chickens and one stall with waterfowl were selected. A purposive sampling approach was used to select the study birds. At each selected LBM stall, blood was collected for serology from 8 apparently healthy live chickens or 7 apparently healthy live ducks, geese, or other waterfowl. For purposes of this study ‘apparently’ healthy status of birds was determined by the local study team veterinarian at the time of LBM visit. Also, at each selected LBM stall, 1 of the 8 apparently healthy chicken or 1 of 7 duck, geese or other waterfowl were purchased at market value, post-mortem (PM) was performed on these birds, and relevant tissue samples were collected for molecular testing. An attempt was made to select birds for PM that were apparently sick (as determined by local team veterinarian at the time of LBM visit). Birds were considered apparently sick if were showing either of the following clinical signs: ruffled feathers, reluctance to move due to weakness, nasal/ or occular discharges, inappetence, severe depression, diarrhoea (whitish, greenish, or yellowish green), or nervous signs (ataxia, wing or limb paralysis). Where apparently sick chickens, ducks or geese were unavailable, apparently healthy chickens, ducks, geese, or other waterfowl were selected for sampling.

### Data collection

#### Selection of stalls

LBM were visited between in November of 2019 across all three states. At each selected LBM, 3 poultry stalls/marketers were purposively selected based on the type of bird species sold (as specified in the inclusion criteria above) and willingness to participate in the study. At each LBM, two stalls with chickens and one stall with waterfowl were selected (Fig. 4).

#### Questionnaire

A structured questionnaire was administered electronically on tablets to LBMs marketers at each market to collect data on socio-demographics of stall owners, characteristics of the stalls and the practices of the stall owners (Supplementary Table S1). The questionnaire was pre-tested prior to administration.

#### Sample collection

At each selected LBM stall, blood and swabs (cloacal, tracheal, and oropharyngeal) were collected from 8 apparently healthy live chickens or 7 apparently healthy live ducks, geese, or other waterfowl. Health status was determined by the local study team veterinarian at the time of visit. At each selected LBM stall, 1 bird was purchased at market value for post-mortem (PM) analysis and tissue samples (lung, spleen, liver and proventriculus) were collected from this bird for molecular testing. This resulted in collection of tissue samples from 2 chickens and 1 waterfowl from each market. The birds undergoing PM were humanely euthanized by a qualified veterinarian using cervical dislocation method. The bird was picked up and the bird’s feet held firmly near the base of their backside and allowed time to calm down if flapping. The chicken’s head was grasped firmly behind the skull between the thumb and the index finger, quickly pulled down and twisted dorsally stretching the neck to separate the skull from the spinal column. This method was chosen because of rapid induction of loss of consciousness and does not chemically contaminate tissues (congestion of organs).

#### Procedures for blood sample collection for serology

Blood samples for serum processing were collected from the brachial (wing) vein. Blood was collected and transferred into vials containing no anticoagulant and kept in a slanted position for 25 min to allow the blood to clot. Serum was separated and placed in 2 ml centrifuge tubes. The tubes were placed in rack in a cooler containing ice packs and transported to the laboratory where detection of serum antibodies was conducted.

#### Procedures for swab and tissue sample collection for PCR

Samples including swabs (cloacal and oropharyngeal swabs) and tissue (lung, spleen, liver, and proventriculus) were collected on FTA cards (Whatman® FTA®). Samples were stored at room temperature and transported to APHA Weybridge for downstream analysis. Swab samples were pooled by species (i.e., chicken or waterfowl) and swab type (i.e., cloacal, or oropharyngeal) in two groups consisting of 4 or 3 individuals for each market stall. For each market, tissue samples were pooled by species and tissue type except for at three markets in Oyo where both species types were pooled together.

### Laboratory testing

#### Detection of antibodies to Avian Paramyxovirus-1 (Newcastle disease)

Serum samples from 405 birds were tested for NDV using the ProFLOK® APMV-1 ELISA kit (Zoetis) following the manufacturer’s recommendations. Sera were stored at −20 °C, and then thawed at room temperature (22 °C – 26 °C) for two hours prior to performing serological assays. The optical density was measured using a Biotek® ELX 800 ELISA Reader with the e-LISA 3.0.0.0 software (© Zoetis).

#### Processing of FTA cards and viral RNA extraction

For assessment of FTA card material for the presence of APMV-1 nucleic acid, samples were recovered in 200 µl of sterile phosphate buffered saline (PBS) by vortexing and incubation at room temperature for 1 h. Following incubation, RNA was extracted from samples using the QIAamp viral RNA mini kit (Qiagen) as per the manufacturer’s instructions. RNA was eluted from QIAamp columns by centrifugation in a final volume of 50 µl [60].

#### Real-time RT-PCR (RRT-PCR) screening

All RNA extracts were screened for generic detection of APMV-1 RNA by rRT-PCR [61]. A positive result using this assay was denoted by a Ct value < 37.0. All plates included a known positive and negative control.

#### Avian Paramyxovirus-1 whole genome sequencing

Ten samples positive for APMV-1 were selected for further analysis by whole genome sequencing (WGS). Samples were selected based on location, species, and presence of a positive APMV-1 RNA signal (Ct value < 32.0). Viral RNA was extracted from selected viral isolates using the QIAmp viral RNA kit (Qiagen, Manchester, UK) without the addition of carrier RNA. Double-stranded cDNA was generated from the RNA using the SuperScript IV First-Strand Synthesis System with random hexamers (ThermoFisher), and the NEBNext Ultra II Non-Directional RNA Second Strand Synthesis Module (New England Biolabs) according to the manufacturer’s instructions and quantified with Quantifluor (Promega, UK). Following cDNA synthesis, the double-stranded cDNA (dsDNA) was purified and concentrated using AMPure XP beads, prior to DNA quantification using the QuantiFluor dsDNA System (Promega) and normalisation of concentration across the samples. Normalised dsDNA (1 ng) was then PCR amplified using the Nextera XT i5 and i7 adapter primers (NexteraXT kit—Illumina, Cambridge, UK). Following PCR amplification, the PCR products were again purified using AMPure XP beads before a second round of quantification and normalisations of the sample concentration before pooling. Pooled sequencing libraries were run on a MiSeq (Illumina, Cambridge, UK) with 2 × 150 base paired end reads. ND F-gene sequences were obtained from the NDV consortium sequence database (https://github.com/NDVconsortium/NDV_Sequence_Datasets). Sequences were aligned using MAFFT v7.48790 [62] and manually trimmed to the F-gene open reading frame using AliView [63]. Phylogenetic trees were then inferred using the maximum-likelihood approach in IQ-Tree v2.1.4 [64] with ModelFinder [65] to infer the appropriate phylogenetic model and 1000 ultrafast bootstraps [66], trees were visualized using Figtree. The APMV-1 whole-genome sequences generated in this study are available through Genbank under the accession numbers: OQ920548, OQ920549, OQ920550, OQ920551, OQ920552, OQ920553, and OQ920554 (Supplementary Table S3).

### Data analyses

The questionnaire data were downloaded from the survey platform, Qualtrics, to Microsoft Excel. Statistical analyses were performed in SPSS Statistics version 25.0 (IBMCorp., Armonk, NY, USA). Descriptive statistics were performed to summarize the LBM stall owner characteristics, serology, and rRT-PCR data. Chi-square test (and/or Fisher exact test and continuity correction for comparisons) were performed to compare proportions of positive samples/birds for selected variables (states, bird types). Analysed variables were considered statistically significant at *p* ≤ 0.05.

## Supplementary Information

Supplementary Material 1

Supplementary Material 2

Supplementary Material 3

## Data Availability

The datasets used and/or analysed during the current study are available from the corresponding author on reasonable request. The APMV-1 whole-genome sequences generated in this study are available through Genbank, under the accession numbers: OQ920548, OQ920549, OQ920550, OQ920551, OQ920552, OQ920553, and OQ920554 (Supplementary Table 3).

## Abbreviations

APMV-1: Avian paramyxovirus type-1
APHA: Animal and Plant Health Agency
Ct: Threshold cycle
ELISA: Enzyme-linked immunosorbent assay
FCT: Federal Capital Territory
LBM: Live bird market
LGACs: Local Government Area Councils
LGAs: Local Government Areas
ND: Newcastle disease
NDV: Newcastle disease virus
PBS: Phosphate buffered saline
PCR: Polymerase chain reaction
PM: Post-mortem
RT-PCR: Reverse transcription polymerase chain reaction
RRT-PCR: Real-time reverse transcription polymerase chain reaction
WGS: Whole genome sequencing

## References

[1] Adene DF OA. The Structure and Importance of the Commercial and Village Based Poultry Industry in Nigeria. Rome: FAO; 2006. Available at: https://www.inter-reseaux.org/wp-content/uploads/pdf_poultrysector_nga_en.pdf

[2] Adene DF OA. Poultry sector country review. Rome: FAO; 2008. Available at: https://www.readkong.com/page/poultry-sector-country-review-fao-animal-production-and-9839281

[3] Organisation FaA. The Future of Livestock in Nigeria. Opportunities and Challenges in the Face of Uncertainty. FAO Rome, Italy; 2019. Available at: https://www.fao.org/3/ca5464en/ca5464en.pdf

[4] Sonaiya E. Family poultry, food security and the impact of HPAI. Worlds Poultry Science Journal. 2007;63(1):132–8. 10.1017/S0043933907001353.

[5] Ameji NO, Oladele OO, Jambalang AR, Adanu AW, Chinyere CN, Meseko CA, et al. Multiple Outbreaks and Clinico-pathological Features of Highly Pathogenic Avian Influenza H5N1 and H5N8 in Poultry Farms in Jos Metropolis, Plateau State. Nigeria Journal of World’s Poultry Research. 2021;11(3):376–86. 10.36380/jwpr.2021.45.

[6] Ekiri A, Armson B, Adebowale K, Endacott I, Galipo E, Alafiatayo R, et al. Evaluating Disease Threats to Sustainable Poultry Production in Africa: Newcastle Disease, Infectious Bursal Disease, and Avian Infectious Bronchitis in Commercial Poultry Flocks in Kano and Oyo States, Nigeria. Front Vet Sci. 2021;8. 10.3389/fvets.2021.730159 10.3389/fvets.2021.730159 PMC8477209 34595231

[7] Alexander D. Newcastle disease and other avian paramyxoviruses. Revue Scientifique et Technique-Office International des Epizooties. Rev Sci Tech. 2000;19(2):443–55. 10.20506/rst.19.2.1231 10.20506/rst.19.2.1231 10935273

[8] Ganar K, Das M, Sinha S, Kumar S. Newcastle disease virus: current status and our understanding. Virus Res. 2014;184:71–81. 10.1016/j.virusres.2014.02.016. 24589707 10.1016/j.virusres.2014.02.016 PMC7127793

[9] Seal BS, King DJ, Bennett JD. Characterization of Newcastle disease virus isolates by reverse transcription PCR coupled to direct nucleotide sequencing and development of sequence database for pathotype prediction and molecular epidemiological analysis. J Clin Microbiol. 1995;33(10):2624–30. 10.1128/jcm.33.10.2624-2630.1995. 8567895 10.1128/jcm.33.10.2624-2630.1995 PMC228544

[10] Panda A, Huang Z, Elankumaran S, Rockemann DD, Samal SK. Role of fusion protein cleavage site in the virulence of Newcastle disease virus. Microb Pathog. 2004;36(1):1–10. 10.1016/j.micpath.2003.07.003. 14643634 10.1016/j.micpath.2003.07.003 PMC7125746

[11] Hill D, Davis OS, Wilde J. Newcastle disease in Nigeria. Br Vet J. 1953;109(9):381–5. 10.1016/S0007-1935(17)50788-5.

[12] Welch C, Shittu I, Abolnik C, Solomon P, Dimitrov K, Taylor T, et al. Genomic comparison of Newcastle disease viruses isolated in Nigeria between 2002 and 2015 reveals circulation of highly diverse genotypes and spillover into wild birds. Arch Virol. 2019;164(8):2031–47. 10.1007/s00705-019-04288-9. 31123963 10.1007/s00705-019-04288-9

[13] Bello M, Yusoff K, Ideris A, Hair-Bejo M, Peeters B, Jibril A, et al. Genotype Diversity of Newcastle Disease Virus in Nigeria: Disease Control Challenges and Future Outlook. Adv Virol. 2018;2018. 10.1155/2018/6097291 10.1155/2018/6097291 PMC6304561 30631359

[14] Snoeck C, Adeyanju A, Owoade A, Couacy-Hymann E, Alkali B, Ottosson U, et al. Genetic Diversity of Newcastle Disease Virus in Wild Birds and Pigeons in West Africa. Appl Environ Microbiol. 2013;79(24):7867–74. 10.1128/AEM.02716-13. 24123735 10.1128/AEM.02716-13 PMC3837833

[15] Snoeck CJ, Ducatez MF, Owoade AA, Faleke OO, Alkali BR, Tahita MC, et al. Newcastle disease virus in West Africa: new virulent strains identified in non-commercial farms. Arch Virol. 2009;154:47–54. 10.1007/s00705-008-0269-5. 19052688 10.1007/s00705-008-0269-5

[16] Van Borm S, Obishakin E, Joannis T, Lambrecht B, van den Berg T. Further evidence for the widespread co-circulation of lineages 4b and 7 velogenic Newcastle disease viruses in rural Nigeria. Avian Pathol. 2012;41(4):377–82. 10.1080/03079457.2012.696311. 22834552 10.1080/03079457.2012.696311

[17] Samuel A, Nayak B, Paldurai A, Xiao S, Aplogan G, Awoume K, et al. Phylogenetic and Pathotypic Characterization of Newcastle Disease Viruses Circulating in West Africa and Efficacy of a Current Vaccine. J Clin Microbiol. 2013;51(3):771–81. 10.1128/JCM.02750-12. 23254128 10.1128/JCM.02750-12 PMC3592067

[18] Cappelle J, Gaidet N, Iverson S, Takekawa J, Newman S, Fofana B, et al. Characterizing the interface between wild ducks and poultry to evaluate the potential of transmission of avian pathogens. Int J Health Geogr. 2011;10. 10.1186/1476-072X-10-60 10.1186/1476-072X-10-60 PMC3280937 22085837

[19] Gowthaman V, Singh SD, Dhama K, Ramakrishnan M, Malik Y, Gopala Krishna Murthy T, et al. Co-infection of Newcastle disease virus genotype XIII with low pathogenic avian influenza exacerbates clinical outcome of Newcastle disease in vaccinated layer poultry flocks. Virus disease. 2019;30:441–52. 10.1007/s13337-019-00533-6 10.1007/s13337-019-00533-6 PMC6864143 31803812

[20] Afonso CL. Virulence during Newcastle disease viruses cross species adaptation. Viruses. 2021;13(1):110. 10.3390/v13010110. 33467506 10.3390/v13010110 PMC7830468

[21] OIE. Newcastle Disease: World Organization for Animal Health; 2022. Available at: https://www.oie.int/en/disease/newcastle-disease.

[22] Chukwudi O, Chukwuemeka E, Mary U. Newcastle Disease Virus Shedding Among Healthy Commercial Chickens and its Epidemiological Importance. Pak Vet J. 2012;32(3):354–6.

[23] Nwanta J, Egege S, Alli-Balogun J, Ezema W. Evaluation of prevalence and seasonality of Newcastle disease in chicken in Kaduna, Nigeria. World‘s Poultry Science Journal. 2008;64(3):416–23. 10.1017/S0043933908000147

[24] Ameji O, Abdu P, Sa’idu L, Isa-Ochepa M. Knowledge of poultry diseases, biosecurity and husbandry practices among stakeholders in poultry production in Kogi State, Nigeria. Sokoto Journal of Veterinary Sciences. 2012;10(2):26–31. 10.4314/sokjvs.v10i2.6

[25] Musa W, Abdu P, Sa’idu L, Bello M. Survey for avian influenza and Newcastle disease antibodies and viruses in domestic and wild birds in Bauchi and Gombe States. Nigeria International Journal of Infectious Diseases. 2016;45:472. 10.1016/j.ijid.2016.02.997.

[26] Shittu I, Joannis TM, Odaibo GN, Olaleye OD. Newcastle disease in Nigeria: epizootiology and current knowledge of circulating genotypes. VirusDisease. 2016;27(4):329–39. 10.1007/s13337-016-0344-6. 28004012 10.1007/s13337-016-0344-6 PMC5142595

[27] Abraham-Oyiguh J, Sulaiman L, Meseko C, Ismail S, Suleiman I, Ahmed S, et al. Prevalence of Newcastle disease antibodies in local chicken in federal capital territory, Abuja, Nigeria. International Scholarly Research Notices. 2014;2014. 10.1155/2014/796148 PMC4897135 27437453

[28] Jibril A, Umoh J, Kabir J, Saidu L, Magaji A, Bello M, et al. Newcastle disease in local chickens of live bird markets and households in Zamfara State, Nigeria. Int Sch Res Notices. 2014;2014. 10.1155/2014/796148

[29] Okoroafor O, Animoke P, Mbegbu E, Aronu C, Nwanta J, Anene B, et al. Prevalence of Newcastle disease virus in feces of free-range turkeys in Enugu. Nigeria Vet World. 2020;13(7):1288–93. 10.14202/vetworld.2020.1288-1293. 32848302 10.14202/vetworld.2020.1288-1293 PMC7429372

[30] Lawal J, El-Yuguda A, Ibrahim U. Survey on prevalence of Newcastle disease antibodies in village poultry at live birds markets in Gombe, Nigeria. J Anim Sci Livest Prod. 2016;1(1):1. 10.21767/2577-0594.100001.

[31] Ameji O, Abdu P, Sa’idu L. Sero-Prevalence of avian influenza, Newcastle and Gumboro disease in chickens in Kogi State, Nigeria. Bulletin of Animal Health and Production in Africa. 2011;59(4):411–8.

[32] Naguib MM, Li R, Ling J, Grace D, Nguyen-Viet H, Lindahl JF. Live and wet markets: food access versus the risk of disease emergence. Trends in Microbiol. 2021;29(7):573–81. 10.1016/j.tim.2021.02.007. 10.1016/j.tim.2021.02.007 PMC9189808 33712334

[33] Oladiran O, Kabir J. Evaluation of poultry processing practices, related public health laws and diseases of chickens at slaughter: A pilot study in Kaduna state. Sokoto Journal of Veterinary Sciences. 2015;13(1):38–47. 10.4314/sokjvs.v13i1.6.

[34] Masaki IvdL MN, Duns H, Toromade F, Ayo O. Poultry Sector Study Nigeria. The Hague: Ministry of Economic Affairs and Climate Policy; 2020 October 2020. Contract No.: RVO 153–2020/RP-INT. Available at: https://www.rvo.nl/sites/default/files/2020/10/Poultry-Sector-Study-Nigeria.pdf

[35] Mihreteab B, Kgotlele T, Neguse F, Petros Y, Habtemariam H, Berhane Y, et al. Phylogenetic analysis of Newcastle disease virus detected in Eritrea between 2017 and 2021. Avian Pathol. 2023;52(6):426–31. 10.1080/03079457.2023.2247370. 37561557 10.1080/03079457.2023.2247370

[36] Amoia CF, Hakizimana JN, Duggal NK, Chengula AA, Rohaim MA, Munir M, et al. Genetic Diversity of Newcastle Disease Virus Involved in the 2021 Outbreaks in Backyard Poultry Farms in Tanzania. Vet Sci. 2023;10(7):477. 10.3390/vetsci10070477. 37505881 10.3390/vetsci10070477 PMC10385779

[37] Tsaxra JB, Abolnik C, Kelly TR, Chengula AA, Mushi JR, Msoffe PLM, et al. Molecular characterization of Newcastle disease virus obtained from Mawenzi live bird market in Morogoro, Tanzania in 2020–2021. Brazilian Journal of Microbiology. 2023;54(4):3265–3273. 10.1007/s42770-023-01159-z. 10.1007/s42770-023-01159-z PMC10689586 37907827

[38] Abolnik C, Horner RF, Bisschop SP, Parker ME, Romito M, Viljoen GJ. A phylogenetic study of South African Newcastle disease virus strains isolated between 1990 and 2002 suggests epidemiological origins in the Far East. Arch Virol. 2004;149(3):603–19. 10.1007/s00705-003-0218-2. 14991446 10.1007/s00705-003-0218-2

[39] Vakuru CT, Kwaghe AV, Kachalla MG, Joannis T, Abdu P, Mshelbwala GM, et al. Investigation of highly pathogenic avian influenza H5N8 outbreak in Kano, Kano State, Nigeria. Int J Sci Eng Res. 2017;8(7):1027–39.

[40] Okwor EC, Eze DC. Epizootic newcastle disease in local chickens reared in south east savannah zone of Nigeria. Int J Poult Sci. 2011;10(3):212–5. 10.3923/ijps.2011.212.215.

[41] Njagi L, Nyaga P, Mbuthia P, Bebora L, Michieka J, Kibe J, et al. Prevalence of Newcastle disease virus in village indigenous chickens in varied agro-ecological zones in Kenya. Livestock Research for Rural Development. 2010;22(5):95. Available at: http://www.lrrd.org/lrrd22/5/njag22095.htm

[42] Ibrahim U, Lawal J, El-Yuguda A. Level of Newcastle disease vaccination awareness and its effects on village chicken production in Gombe State, Nigeria. Direct Research Journal of Agriculture and Food Science. 2016;4(3):48–54. Available at: https://directresearchpublisher.org/drjafs/files/2016/02/DRJA10907278.pdf

[43] Aliyu H, Sa’idu L, Abdu P, Oladele S. Retrospective analysis of Newcastle disease diagnosed at the poultry clinic of Ahmadu Bello University, Zaria. Nigeria Sokoto Journal of Veterinary Sciences. 2015;13(3):42–8. 10.4314/SOKJVS.V13I3.7.

[44] Ogungbenro SB, Morakinyo TE. Rainfall distribution and change detection across climatic zones in Nigeria. Weather and Climate Extremes. 2014;5:1–6. 10.1016/j.wace.2014.10.002.

[45] Njoga EO, Ogugua AJ, Nwankwo IO, Awoyomi OJ, Okoli CE, Buba DM, et al. Antimicrobial drug usage pattern in poultry farms in Nigeria: implications for food safety, public health and poultry disease management. Vet Ital. 2021;57(1):5–12. 10.12834/VetIt.2117.11956.1. 34313093 10.12834/VetIt.2117.11956.1

[46] Jeon W-J, Lee E-K, Lee Y-J, Jeong O-M, Kim Y-J, Kwon J-H, et al. Protective efficacy of commercial inactivated Newcastle disease virus vaccines in chickens against a recent Korean epizootic strain. J Vet Sci. 2008;9(3):295–300. 10.4142/jvs.2008.9.3.295. 18716450 10.4142/jvs.2008.9.3.295 PMC2811842

[47] Spackman E, Pedersen JC, McKinley ET, Gelb J. Optimal specimen collection and transport methods for the detection of avian influenza virus and Newcastle disease virus. BMC Vet Res. 2013;9(1):1–12. 10.1186/1746-6148-9-35. 23432911 10.1186/1746-6148-9-35 PMC3599916

[48] Panus A, Setiyaningsih S, Mayasari N. Newcastle disease virus infection study on duck and chicken in Subang district. Jurnal Ilmu Ternak dan Veteriner. 2015;20:134–47. 10.14334/JITV.V20I2.1168.

[49] Susta L, Segovia D, Olivier TL, Dimitrov KM, Shittu I, Marcano V, et al. Newcastle disease virus infection in quail. Vet Pathol. 2018;55(5):682–92. 10.1177/0300985818767996. 29661124 10.1177/0300985818767996

[50] Das A, Spackman E, Pantin-Jackwood MJ, Suarez DL. Removal of real-time reverse transcription polymerase chain reaction (RT-PCR) inhibitors associated with cloacal swab samples and tissues for improved diagnosis of Avian influenza virus by RT-PCR. J Vet Diagn Invest. 2009;21(6):771–8. 10.1177/104063870902100603. 19901277 10.1177/104063870902100603

[51] Perozo F, Villegas P, Estevez C, Alvarado I, Purvis LB. Use of FTA® filter paper for the molecular detection of Newcastle disease virus. Avian Pathol. 2006;35(02):93–8. 10.1080/03079450600597410. 16595299 10.1080/03079450600597410

[52] Cardona-Ospinaa J, Villalba-Mirandaa MF, Palechor-Ocampoa LA, Mancillaa LI, Sepúlveda-Ariasa JC. A systematic review of FTA cards® as a tool for viral RNA preservation in fieldwork: Are they safe and effective? Prev Vet Med. 2019;172: 104772. 10.1016/j.prevetmed.2019.104772. 31607414 10.1016/j.prevetmed.2019.104772 PMC7126379

[53] Davis EH, Velez JO, Russell BJ, Basile AJ, Brault AC, Hughes HR. Evaluation of Whatman FTA cards for the preservation of yellow fever virus RNA for use in molecular diagnostics. PLoS Negl Trop Dis. 2022;16(12): e0011027. 10.1371/journal.pntd.0010487 10.1371/journal.pntd.0010487 PMC9200311 35704565

[54] Daodu OB, Aiyedun JO, Kadir RA, Ambali HM, Oludairo OO, Olorunshola ID, et al. Awareness and antibody detection of Newcastle disease virus in a neglected society in Nigeria. Vet World. 2019;12(1):112–8. 10.14202/vetworld.2019.112-118. 30936663 10.14202/vetworld.2019.112-118 PMC6431813

[55] Anzaku S, Umoh J, Abdu P, Kabir J, Bala A. Serological Survey of Newcastle Disease in Free Ranging Local Chickens in the Federal Capital Territory, Abuja, Nigeria. New Journal of Science. 2017, Article ID 9646138. 10.1155/2017/9646138

[56] Ameh JA, Mailafia S, Olabode OH, Adah BJ, Ogbole ME, Alalade DI. Sero-prevalence of Newcastle disease virus antibodies in local and exotic chickens in Gwagwalada, Nigeria. Journal of Veterinary Medicine and Animal Health. 2016;8(11):193–8. 10.5897/JVMAH2016.0482.

[57] Liverpool-Tasie LSO, Omonona B, Sanou A, Ogunleye W, Padilla S, Reardon T. Growth and transformation of food systems in Africa: evidence from the poultry value chain in Nigeria. Feed the Future Innovation Lab for Food Security Policy Research Briefs 260417, Michigan State University, Department of Agricultural, Food, and Resource Economics, Feed the Future Innovation Lab for Food Security (FSP). 10.22004/ag.econ.260417

[58] Thrushfield MV. Veterinary Epidemiology. 3rd ed. Oxford (UK): Blackwell Publishing; 2005.

[59] Waziri NE, Nguku P, Olayinka A, Ajayi I, Kabir J, Okolocha E, et al. Evaluating a surveillance system: live-bird market surveillance for highly pathogenic avian influenza, a case study. Pan Afr Med J. 2014;18 Suppl 1(Suppl 1):11. 10.11694/pamj.supp.2014.18.1.4188. 10.11694/pamj.supp.2014.18.1.4188 PMC4199346 25328630

[60] Slomka MJ, Pavlidis T, Coward VJ, Voermans J, Koch G, Hanna A, et al. Validated RealTime reverse transcriptase PCR methods for the diagnosis and pathotyping of Eurasian H7 avian influenza viruses. Influenza Other Respir Viruses. 2009;3(4):151–64. 10.1111/j.1750-2659.2009.00083.x. 19627372 10.1111/j.1750-2659.2009.00083.x PMC4634683

[61] Sutton DA, Allen DP, Fuller CM, Mayers J, Mollett BC, Londt BZ, et al. Development of an avian avulavirus 1 (AAvV-1) L-gene real-time RT-PCR assay using minor groove binding probes for application as a routine diagnostic tool. J Virol Methods. 2019;265:9–14. 10.1016/j.jviromet.2018.12.001. 30579921 10.1016/j.jviromet.2018.12.001

[62] Katoh K, Toh H. Parallelization of the MAFFT multiple sequence alignment program. Bioinformatics. 2010;26(15):1899–900. 10.1093/bioinformatics/btq224. 20427515 10.1093/bioinformatics/btq224 PMC2905546

[63] Larsson A. AliView: a fast and lightweight alignment viewer and editor for large datasets. Bioinformatics. 2014;30(22):3276–8. 10.1093/bioinformatics/btu531. 25095880 10.1093/bioinformatics/btu531 PMC4221126

[64] Minh BQ, Schmidt HA, Chernomor O, Schrempf D, Woodhams MD, Von Haeseler A, et al. IQ-TREE 2: new models and efficient methods for phylogenetic inference in the genomic era. Mol Biol Evol. 2020;37(5):1530–4. 10.1093/molbev/msaa015. 32011700 10.1093/molbev/msaa015 PMC7182206

[65] Kalyaanamoorthy S, Minh BQ, Wong TKF, von Haeseler A, Jermiin LS. ModelFinder: fast model selection for accurate phylogenetic estimates. Nat Methods. 2017;14(6):587–9. 10.1038/nmeth.4285. 28481363 10.1038/nmeth.4285 PMC5453245

[66] Hoang D, Chernomor O, Von Haeseler A. UFBoot2: Improving the ultrafast bootstrap approximation. Mol Biol Evol. 2018;35:518–22. 10.1093/molbev/msx281. 29077904 10.1093/molbev/msx281 PMC5850222

