# Supplementary material for: Evaluating the impact of avian paramyxovirus type 1 infection in poultry at live bird markets in Nigeria: defining hurdles to sustainable agriculture
Source: BMC Vet Res. 2025 Feb 12;21:62. doi: 10.1186/s12917-025-04508-2 (PMC11817539; doi:10.1186/s12917-025-04508-2)
Supplement: Supplementary file 1 — Supplementary Material 1 [file 12917_2025_4508_MOESM1_ESM.pdf]

Supplementary Table S1. Characteristics of the owners of the surveyed stalls in total and by state.

Stall owner characteristics	Total (n=54)	Kano (n=18)	Oyo (n=17)	FCT (Abuja) (n=19)	*P* for difference by state *
	n (%)	n (%)	n (%)	n (%)	
**Gender**					**<0.001**[1]
Male	39 (72.2%)	18 (100%)	2 (11.8%)	19 (100%)	
Female	15 (27.8%)	0 (0%)	15 (88.2%)	0 (0%)	
**Age**					n/a [2]
< 35 years	11 (20.4%)	3 (16.7%)	6 (35.3%)	2 (10.5%)	
35-45 years	16 (29.6%)	4 (22.2%)	2 (11.8%)	10 (52.6%)	
46-60 years	20 (37.0%)	6 (33.3%)	7 (41.2%)	7 (36.8%)	
> 60 years	7 (13.0%)	5 (27.8%)	2 (11.8%)	0 (0%)	
**Education level**					n/a [2]
None	14 (25.9%)	3 (16.7%)	5 (29.4%)	6 (31.6%)	
Primary	9 (16.7%)	4 (22.2%)	2 (11.8%)	3 (15.8%)	
Secondary	21 (38.9%)	10 (55.6%)	7 (41.2%)	4 (21.1%)	
Diploma certificate	5 (9.3%)	1 (5.6%)	1 (5.9%)	3 (15.8%)	
Degree or higher	5 (9.3%)	0 (0%)	2 (11.8%)	3 (15.8%)	
**Registration status**					n/a [2]
Not registered marketer	2 (3.7%)	1 (5.6%)	0 (0%)	1 (5.3%)	
Registered marketer [3]	52 (96.3%)	17 (94.4%)	17 (100%)	18 (94.7%)	

[*] *P*-values from Chi-square tests. [1] *P*-value from a Fisher's Exact Test was reported due to cells with an expected count < 5. [2] *P*-values were not reported as the Chi-square test was not valid due to a high number of cells with an expected count < 5. [3] All registered owners were registered with the state live bird market.

Abbreviations: FCT, Federal Capital Territory; n/a, not available.
