# Supplementary material for: Evaluating the impact of avian paramyxovirus type 1 infection in poultry at live bird markets in Nigeria: defining hurdles to sustainable agriculture
Source: BMC Vet Res. 2025 Feb 12;21:62. doi: 10.1186/s12917-025-04508-2 (PMC11817539; doi:10.1186/s12917-025-04508-2)
Supplement: Supplementary file 2 — Supplementary Material 2 [file 12917_2025_4508_MOESM2_ESM.pdf]

Supplementary Table S2. Characteristics of the surveyed stalls and the practices of the stall owners in total and by state.

Stall characteristics and practices of the stall owners	Total (n=54)	Kano (n=18)	Oyo (n=17)	FCT (Abuja) (n=19)	*P* for difference by state *
	n (%)	n (%)	n (%)	n (%)	
**Live bird market (LBM)**					
Separated from other market sections (if yes)	43 (79.6%)	10 (55.6%)	14 (82.4%)	19 (100%)	n/a
Presence/evidence of rodents (if yes)	32 (59.3%)	14 (77.8%)	0 (0%)	18 (94.7%)	**<0.001**
Presence/evidence of wild birds (if yes)	12 (22.2%)	6 (33.3%)	0 (0%)	6 (31.6%)	n/a
**Cage type**					
Wooden	53 (98.1%)	17 (94.4%)	17 (100%)	19 (100%)	n/a
Wire-mesh	48 (88.9%)	17 (94.4%)	12 (70.6%)	19 (100%)	n/a
Plastic	1 (1.9%)	0 (0%)	1 (5.9%)	0 (0%)	n/a
Other [1]	1 (1.9%)	1 (5.6%)	0 (0%)	0 (0%)	n/a
**Poultry type sold at stall**					
Chickens	50 (92.6%)	18 (100%)	13 (76.5%)	19 (100%)	n/a
Ducks/geese	33 (61.1%)	9 (50.0%)	5 (29.4%)	19 (100%)	**<0.001**
Turkeys	35 (64.8%)	11 (61.1%)	5 (29.4%)	19 (100%)	**<0.001**
Quails	9 (16.7%)	1 (5.6%)	1 (5.9%)	7 (36.8%)	n/a
Guinea fowl	35 (64.8%)	12 (66.7%)	4 (23.5%)	19 (100%)	**<0.001**
Pigeons	32 (59.3%)	11 (61.1%)	2 (11.8%)	19 (100%)	**<0.001**
Other [2]	3 (5.6%)	2 (11.1%)	1 (5.9%)	0 (0%)	n/a
**Average number of birds for sale daily**					n/a
< 20 birds	8 (14.8%)	1 (5.6%)	6 (35.3%)	1 (5.3%)	
20-50 birds	15 (27.8%)	7 (38.9%)	8 (47.1%)	0 (0%)	
51-100 birds	8 (14.8%)	3 (16.7%)	3 (17.6%)	2 (10.5%)	
>100 birds	23 (42.6%)	7 (38.9%)	0 (0%)	16 (84.2%)	
**Average number of birds sold daily**					n/a
< 20 birds	17 (31.5%)	4 (22.2%)	12 (70.6%)	1 (5.3%)	
21-40 birds	11 (20.4%)	7 (38.9%)	2 (11.8%)	2 (10.5%)	
41-60 birds	4 (7.4%)	3 (16.7%)	1 (5.9%)	0 (0%)	
61-80 birds	2 (3.7%)	0 (0%)	1 (5.9%)	1 (5.3%)	
81-100 birds	2 (3.7%)	2 (11.1%)	0 (0%)	0 (0%)	
>100 birds	18 (33.3%)	2 (11.1%)	1 (5.9%)	15 (78.9%)	
**Bird source**					
Village households	33 (61.1%)	18 (100%)	3 (17.6%)	12 (63.2%)	**<0.001**
Commercial farms	36 (66.7%)	10 (55.6%)	10 (58.8%)	16 (84.2%)	0.141
Self-raised	11 (20.4%)	3 (16.7%)	0 (0%)	8 (42.1%)	n/a
Mobile sellers/middle-men	14 (25.9%)	3 (16.7%)	1 (5.9%)	10 (52.6%)	n/a
Other LBM inside state	8 (14.8%)	3 (16.7%)	1 (5.9%)	4 (21.1%)	n/a
Other LBM outside state	10 (18.5%)	0 (0%)	4 (23.5%)	6 (31.6%)	n/a
Other [3]	1 (1.9%)	0 (0%)	1 (5.9%)	0 (0%)	n/a
**Bird-feed type**					
Commercial	36 (66.7%)	8 (44.4%)	11 (64.7%)	17 (89.5%)	**0.014**
Locally compounded	36 (66.7%)	18 (100%)	6 (35.3%)	12 (63.2%)	**<0.001**
Other	0 (0%)	n/a	n/a	n/a	n/a

Supplementary Table S2. Continued.

Stall characteristics and practices of the stall owners	Total (n=54)	Kano (n=18)	Oyo (n=17)	FCT (Abuja) (n=19)	*P* for difference by state *
	n (%)	n (%)	n (%)	n (%)	
**Bird-feed source**					
Purchased from other sellers	43 (79.6%)	10 (55.6%)	16 (94.1%)	17 (89.5%)	n/a
Self-produced	28 (51.9%)	16 (88.9%)	1 (5.9%)	11 (57.9%)	**<0.001**
Other	0 (0%)	n/a	n/a	n/a	n/a
**Water source**					
Piped	24 (44.4%)	6 (33.3%)	6 (35.3%)	12 (63.2%)	0.151
Streamed	1 (1.9%)	0 (0%)	1 (5.9%)	0 (0%)	n/a
Well	8 (14.8%)	0 (0%)	8 (47.1%)	0 (0%)	n/a
Borehole	31 (57.4%)	14 (77.8%)	8 (47.1%)	9 (47.4%)	0.115
Other	0 (0%)	n/a	n/a	n/a	n/a
**Ways to ensure newly bought birds are healthy**					
Know and trust supplier	1 (1.9%)	1 (5.6%)	0 (0%)	0 (0%)	n/a
Visual check	53 (98.1%)	17 (94.4%)	17 (100%)	19 (100%)	n/a
Other [4]	5 (9.3%)	5 (27.8%)	0 (0%)	0 (0%)	n/a
**Old and newly bought birds are separated**					n/a
Never	10 (18.5%)	4 (22.2%)	6 (35.3%)	0 (0%)	
Sometimes	4 (7.4%)	1 (5.6%)	3 (17.6%)	0 (0%)	
Always	40 (74.1%)	13 (72.2%)	8 (47.1%)	19 (100%)	
**Ways to separate newly bought birds from old**					
Separate cages	44 (81.5%)	14 (77.8%)	11 (64.7%)	19 (100%)	n/a
Other	0 (0%)	n/a	n/a	n/a	n/a
**Unsold birds keeping location**					
At the market	49 (90.7%)	17 (94.4%)	13 (76.5%)	19 (100%)	n/a
Home/own farm	4 (7.4%)	0 (0%)	4 (23.5%)	0 (0%)	n/a
Other [5]	1 (1.9%)	1 (5.6%)	0 (0%)	0 (0%)	n/a
**Owner has own poultry at home/farm (if yes)**	17 (31.5%)	6 (33.3%)	2 (11.8%)	9 (47.4%)	0.070
**Owner sells birds at other LBMs (if yes)**	19 (35.2%)	4 (22.2%)	7 (41.2%)	8 (42.1%)	0.369
**Frequency of sick/dead birds at stall**					n/a
Never	2 (3.7%)	0 (0%)	2 (11.8%)	0 (0%)	
Rarely (1/month)	20 (37.0%)	6 (33.3%)	11 (64.7%)	3 (15.8%)	
Sometimes (2-3/week)	26 (48.1%)	11 (61.1%)	3 (17.6%)	12 (63.2%)	
Frequently (at least 1/day)	6 (11.1%)	1 (5.6%)	1 (5.9%)	4 (21.1%)	
**Procedures for sick birds at stall**					
Isolation	39 (72.2%)	17 (94.4%)	3 (17.6%)	19 (100%)	**<0.001** **
Treatment	43 (79.6%)	13 (72.2%)	13 (76.5%)	17 (89.5%)	n/a
Report to vet/clinic	19 (35.2%)	3 (16.7%)	0 (0%)	16 (84.2%)	**<0.001**
Other [6]	3 (5.6%)	2 (11.1%)	1 (5.9%)	0 (0%)	n/a

Supplementary Table S2. Continued.

Stall characteristics and practices of the stall owners	Total (n=54)	Kano (n=18)	Oyo (n=17)	FCT (Abuja) (n=19)	*P* for difference by state *
	n (%)	n (%)	n (%)	n (%)	
**Ways of getting rid of sick birds**					
Sell	6 (11.1%)	2 (11.1%)	1 (5.9%)	3 (15.8%)	n/a
Slaughter for meat consumption	38 (70.4%)	17 (94.4%)	13 (76.5%)	8 (42.1%)	**0.001**
Feed to dogs/cats	0 (0%)	n/a	n/a	n/a	n/a
Dispose (bury/burn)	10 (18.5%)	0 (0%)	3 (17.6%)	7 (36.8%)	n/a
Other [7]	4 (7.4%)	0 (0%)	0 (0%)	4 (21.1%)	n/a
**Owner reports dead birds (if yes)**	19 (35.2%)	2 (11.1%)	0 (0%)	17 (89.5%)	**<0.001**
Reported to government veterinarian	2 (3.7%)	0 (0%)	n/a	2 (10.5%)	n/a
Reported to state veterinarian	16 (29.6%)	1 (5.6%)	n/a	15 (78.9%)	n/a
Reported to other [8]	1 (1.9%)	1 (5.6%)	n/a	0 (0%)	n/a
**Ways of disposing of dead birds**					
Waste dump site	32 (59.3%)	13 (72.2%)	9 (52.9%)	10 (52.6%)	0.391
Bury/burn	24 (44.4%)	12 (66.7%)	1 (5.9%)	11 (57.9%)	**<0.001**
Meat consumption	6 (11.1%)	0 (0%)	6 (35.3%)	0 (0%)	n/a
Feed dogs/cats	6 (11.1%)	2 (11.1%)	2 (11.8%)	2 (10.5%)	n/a
Other	0 (0%)	n/a	n/a	n/a	n/a
**Ways of disposing of waste**					
Waste dump site	21 (38.9%)	4 (22.2%)	14 (82.4%)	3 (15.8%)	**<0.001**
Bury/burn	4 (7.4%)	2 (11.1%)	0 (0%)	2 (10.5%)	n/a
Sell	23 (42.6%)	8 (44.4%)	3 (17.6%)	12 (63.2%)	**0.022**
Other [9] (mostly used as manure)	9 (16.7%)	6 (33.3%)	0 (0%)	3 (15.8%)	n/a
**Owner transports poultry from different sources together (if yes)**	20 (37.0%)	4 (22.2%)	11 (64.7%)	5 (26.3%)	**0.016**
**Owner has own transport (if yes)**	12 (22.2%)	3 (16.7%)	3 (17.6%)	6 (31.6%)	n/a
**Transport used for poultry**					
Private car/vehicle	11 (20.4%)	1 (5.6%)	4 (23.5%)	6 (31.6%)	n/a
Public car/vehicle	42 (77.8%)	15 (83.3%)	13 (76.5%)	14 (73.7%)	n/a
Private motorcycle	1 (1.9%)	1 (5.6%)	0 (0%)	0 (0%)	n/a
Public motorcycle	1 (1.9%)	1 (5.6%)	0 (0%)	0 (0%)	n/a
Bicycle	0 (0%)	n/a	n/a	n/a	n/a
Walk and carry	0 (0%)	n/a	n/a	n/a	n/a
Other	0 (0%)	n/a	n/a	n/a	n/a
**Ways owner carries poultry to be sold**					
In baskets	34 (63.0%)	7 (38.9%)	9 (52.9%)	18 (94.7%)	**0.001**
In cages/crates	29 (53.7%)	4 (22.2%)	6 (35.3%)	19 (100 %)	**<0.001**
Tied and piled loosely	13 (24.1%)	8 (44.4%)	5 (29.4%)	0 (0%)	n/a
Other [10]	2 (3.7%)	2 (11.1%)	0 (0%)	0 (0%)	n/a
**Frequency of cleaning the vehicle for poultry transport after each use**					n/a
Never	3 (5.6%)	3 (16.7%)	0 (0%)	0 (0%)	
Sometimes	6 (11.1%)	2 (11.1%)	4 (23.5%)	0 (0%)	
Always	45 (83.3%)	13 (72.2%)	13 (76.5%)	19 (100%)	

Supplementary Table S2. Continued.

Stall characteristics and practices of the stall owners	Total (n=54)	Kano (n=18)	Oyo (n=17)	FCT (Abuja) (n=19)	*P* for difference by state *
	n (%)	n (%)	n (%)	n (%)	
**Frequency of cleaning premises with bird cages/stalls**					n/a
Never	1 (1.9%)	1 (5.6%)	0 (0%)	0 (0%)	
1/month	3 (5.6%)	1 (5.6%)	2 (11.8%)	0 (0%)	
2-3/month	2 (3.7%)	0 (0%)	2 (11.8%)	0 (0%)	
1/week	5 (9.3%)	0 (0%)	4 (23.5%)	1 (5.3%)	
Several times/week	43 (79.6%)	16 (88.9%)	9 (52.9%)	18 (94.7%)	
**Frequency of cleaning cages/baskets after each use with soap and water**					n/a
Never	2 (3.7%)	2 (11.1%)	0 (0%)	0 (0%)	
Sometimes	19 (35.2%)	13 (72.2%)	6 (35.3%)	0 (0%)	
Always	33 (61.1%)	3 (16.7%)	11 (64.7%)	19 (100%)	
**Frequency of cleaning cages/baskets after each use with disinfectant**					n/a
Never	6 (11.1%)	3 (16.7%)	0 (0%)	3 (15.8%)	
Sometimes	31 (57.4%)	14 (77.8%)	13 (76.5%)	4 (21.0%)	
Always	17 (31.5%)	1 (5.6%)	4 (23.5%)	12 (63.2%)	
**Frequency of washing hands after handling poultry**					n/a
Never	1 (1.9%)	0 (0%)	1 (5.9%)	0 (0%)	
Sometimes	13 (24.1%)	4 (22.2%)	9 (52.9%)	0 (0%)	
Always	40 (74.1%)	14 (77.8%)	7 (41.2%)	19 (100%)	
**Ways of hand-washing [11]**					n/a
Soap and water	39 (73.6%)	15 (83.3%)	6 (37.5%)	18 (94.7%)	
Water only	13 (24.5%)	3 (16.7%)	10 (62.5%)	0 (0%)	
Water and disinfectant	1 (1.9%)	0 (0%)	0 (0%)	1 (5.3%)	
Other	0 (0%)	n/a	n/a	n/a	

[*]*P*-values from Chi-square tests. [**] *P*-value from a Fisher's Exact Test was reported due to cells with an expected count < 5. [1] Other includes: wooden cages with wire-mesh (n=1). [2] Other includes: local chicken (n=1), peacock (n=1) and rabbits (n=1). [3] Other includes: village farms (n=1). [4] Other includes: experience based (n=1), palpation (n=1), physical examination (n=1), signs observed (n=1) and thriftiness, mucus membrane, comb, wattle changes (n=1). [5] Other includes: placed in separate room (n=1). [6] Other includes: salvage at times (n=1), slaughter for consumption (n=2). [7] Other includes: isolate and treat (n=2), keep in isolated unit where they are treated (n=1) and none (n=1). [8] Other includes: private laboratory (n=1). [9] Other includes: farmers collect them for their farms (n=1), given to crop farmers as manure (n=1), taken to farm (n=1), taken to farm as manure (n=4), used as manure (n=1) and used to fertilize my farm (n=1). [10] Other includes: inside packages (n=1) and placed in the car without tying (n=1). [11] These data were available for n=53 stalls. n/a, *P*-values were not reported as the Chi-square test was not valid due to a high number of cells with an expected count < 5. Abbreviations: FCT, Federal Capital Territory; LBM, live bird market; n/a, not available or applicable
