# Supplementary material for: Evaluating the impact of avian paramyxovirus type 1 infection in poultry at live bird markets in Nigeria: defining hurdles to sustainable agriculture
Source: BMC Vet Res. 2025 Feb 12;21:62. doi: 10.1186/s12917-025-04508-2 (PMC11817539; doi:10.1186/s12917-025-04508-2)
Supplement: Supplementary file 3 — Supplementary Material 3 [file 12917_2025_4508_MOESM3_ESM.pdf]

Supplementary Table S3. Samples selected for whole genome sequencing from APMV-1 rRT-PCR positive clinical samples collected in November 2019 from poultry at live bird markets in Nigeria.

Sample ID	State	Species	Ct Value*	Strain[1]	Accession Number
F0101C8S	Kano	Chicken	22.6	APMV-1/Chicken/Nigeria/F0101C8S/2019	OQ920548
F0201C8P	Kano	Chicken	21.7	APMV-1/Chicken/Nigeria/F0201C8P/2019	OQ920549
F0301C8P	Kano	Chicken	22.6	APMV-1/Chicken/Nigeria/F0301C8P/2019	OQ920550
F1402C1OS	FCT (Abuja)	Chicken	31.4	APMV-1/Chicken/Nigeria/F1402C1OS/2019	OQ920551
F1801C8S	FCT (Abuja)	Chicken	20.1	APMV-1/Chicken/Nigeria/F1801C8S/2019	OQ920552
F0601C8LU	Kano	Chicken	27.5	APMV-1/Chicken/Nigeria/F0601C8LU/2019	OQ920553
F1701C8S	FCT (Abuja)	Domestic duck	31.8	APMV-1/Waterfowl/Nigeria/F1701C8S/2019	OQ920554
F0403W5OS	Kano	Domestic duck	30.7	No NGS	*n/a*
F0703W1OS	Oyo	Domestic duck	33	No NGS	*n/a*
F1803W7S	FCT (Abuja)	Domestic duck	30.5	No NGS	*n/a*

*Ct values from rRT-PCR targeting APMV-1 L-Gene. [1]Strain names assigned for samples with sequencing data, samples where sequencing failed indicated by ‘No NGS’. Abbreviations: FCT, Federal Capital Territory; n/a, not applicable.
